# An exploratory study on spatiotemporal clustering of suicide in Korean adolescents

**DOI:** 10.1186/s13034-024-00745-9

**Published:** 2024-05-10

**Authors:** Won-Seok Choi, Beop-Rae Roh, Duk-In Jon, Vin Ryu, Yunhye Oh, Hyun Ju Hong

**Affiliations:** 1 Department of Psychiatry, Yeouido St. Mary’s Hospital, College of Medicine, The Catholic University of Korea, Seoul, Republic of Korea; 2 Department of Social Welfare, Pukyong National University, Busan, Republic of Korea; 3 Department of Psychiatry, Hallym University Sacred Heart Hospital, Hallym University, 22, Gwanpyeong-ro 170Beon-gil, Dongan-gu, Anyang, Gyeonggi-do Republic of Korea; 4 Hallym University Suicide and School Mental Health Institute, Anyang, Republic of Korea

**Keywords:** Adolescent, Suicide, Suicide cluster, Space–time, Density-based clustering, Korea

## Abstract

**Background:**

Adolescent suicides are more likely to form clusters than those of other age groups. However, the definition of a cluster in the space–time dimension has not been established, neither are the factors contributing to it well known. Therefore, this study aimed to identify space–time clusters in adolescent suicides in Korea and to examine the differences between clustered and non-clustered cases using novel statistical methods.

**Methods:**

From 2016 to 2020, the dates and locations, including specific addresses from which the latitude and longitude of all student suicides (aged 9–18 years) in Korea were obtained through student suicide reports. Sociodemographic characteristics of the adolescents who died by suicide were collected, and the individual characteristics of each student who died by suicide were reported by teachers using the Strengths and Difficulties Questionnaire (SDQ). Density-Based Spatial Clustering of Applications with Noise (DBSCAN) analysis was used to assess the clustering of suicides.

**Results:**

We identified 23 clusters through the data analysis of 652 adolescent suicides using DBSCAN. By comparing the size of each cluster, we identified 63 (9.7%) spatiotemporally clustered suicides among adolescents, and the temporal range of these clusters was 7–59 days. The suicide cluster group had a lower economic status than the non-clustered group. There were no significant differences in other characteristics between the two groups.

**Conclusion:**

This study has defined the space–time cluster of suicides using a novel statistical method. Our findings suggest that when an adolescent suicide occurs, close monitoring and intervention for approximately 2 months are needed to prevent subsequent suicides. Future research using DBSCAN needs to involve a larger sample of adolescents from various countries to further corroborate these findings.

## Introduction

Suicide is a global social issue in adolescents aged 15–19 years old, for whom it was the fourth leading cause of death globally in 2019 [1]. This makes it a great social burden. Post the advent of the COVID-19 pandemic in 2020, there has been a global increase in suicide attempts and suicidal ideation among youth, making it an even more critical issue today [2]. Suicide in adolescents is heterogeneous and distinguished from the suicide of adults by complicated factors, including family, school, and individual components [3].

Suicide incidents do not always occur randomly; sometimes, they occur in clusters. This phenomenon has been described as ‘contagion’ or ‘clustering of suicide.’ Although the two words are often used interchangeably, “contagion” was considered as a mechanism of “clustering of suicide” and more recently, “social transmission” is regarded as a narrower and more explicit mechanism for clustering [4–6]. Two main types of suicide clusters are argued in the previous study—mass clusters, which is a media-related phenomenon that suicide rates increase in a wide population in a time period, and space–time clusters, where suicides occur in unusually concentrated within a specific locality of time and space [6, 7]. Clinically, space–time clustered suicide may refer to suicides influenced by the suicide of someone around them, such as a friend. Previous studies have shown that suicides of 15–24 years of age are more likely to cluster than other age groups [8] and account for 1–6% of suicides among youth [8–11]. Temporal and spatial definitions are useful in terms of suicide prevention. If a youth suicide occurs, more close monitoring of follow-up suicides, management of risk factors, and crisis intervention during the period and legion corresponding to the cluster may contribute to suicide prevention.

Since the clustering of suicide began to be discussed in the clinical field approximately 40 years ago [12, 13], several statistical techniques for detecting and defining of space–time clusters of suicide has been used to detect and define space–time clusters of suicide [6, 8, 10, 11, 14–18]. However, there is currently no specific definition or gold standard for detecting suicide clusters [5, 17, 18].

The Knox procedure, used in earlier studies, considers all possible pairs of suicide cases and the temporal and spatial distances between them. This method established clustering by demonstrating a positive relationship between the temporal and spatial distances of a pair. The Knox method requires the specification of critical values of time and space to define closeness, and previous studies have set the county level spatially and 7, 14, 30 and 60 days temporally [8, 14, 15].

Scan statistics represents a more advanced method than the Knox procedure. It investigates clustering within a variable time window across varying geographical areas and compares the expected number of cases and actual number of cases inside and outside the scanning window [10, 19–21]. The results of this type of analysis are a set of cylinders, where the base represents the area of the potential cluster, and the height represents the time period of the cluster. Previous studies analyzed the presence of clustered by setting a specific window of various ranges and a temporal window from 7 days to 2 years [10, 11, 17, 22–25]. However, previous studies using scan statistics have some limitations, primarily in their focus on detecting clusters with a circular shape [26] and its focus on larger spatial regions, such as those represented in county-level data [11, 17, 22, 23, 27–29].

In terms of the analytic method, previous studies have defined spatiotemporal parameters in advance and somewhat arbitrarily based on the researchers’ judgment, resulting in the clusters of suicides showing spatiotemporal closeness being confirmed. For example, the temporal parameters were set to 7, 14, 30, and 60 days [8, 14, 15], but suicide clusters could occur outside this window. Therefore, identifying the more sensitive periods for suicide clusters is an important research objective.

Several previous studies targeting the entire population, including adolescents and young adults, have compared the characteristics of clustered and non-clustered suicides and reported that clustering was more common among young men than women [23, 30], those living in rural areas, [23, 25, 30, 31], and those experiencing economic deprivation [31]. However, when narrowing the target population to include only adolescents, one study found no definite differences in clinical characteristics between the suicide cluster and non-cluster groups [5], while several studies reported that the suicide cluster group had a lower economic level and included more adolescent boys than the non-cluster groups [5, 6, 18].

A new analytical method using machine learning [32, 33] that does not preset spatiotemporal parameters with a narrower unit of spatiotemporal data of adolescents can increase the understanding of the space–time clusters in adolescent suicide, which is not well known.

In Korea, adolescent suicide is a serious social problem and is the leading cause of death among young people aged 10–19 years [34]. In particular, during the COVID-19 pandemic, the suicide rate among adolescents increased at a faster rate than that of older adults [35]. The suicide rate among adolescents was higher after the pandemic than before [35], reaching 9.5 per 100,000 in 2021 for adolescents aged 15–17 years, compared to 5.8 per 100,000 in 2017 and 7.5 per 100,000 in 2018 [36].

This study analyzed an entire dataset of students who died by suicide from 2016 to 2020 that was collected through the Korean Ministry of Education and included the date of death and the specific address from which latitude and longitude coordinates can be extracted. We hypothesized that there would be space–time clusters of suicides among Korean adolescents, and that if clustered and non-clustered suicides were distinguishable, there would be differences in their characteristics. This study will contribute to suicide prevention efforts by identifying the critical period in which subsequent suicides are most likely.

## Methods

### Database

This study used data from student suicide reports collected by the Korean Ministry of Education from January 1, 2016, to December 31, 2020. In Korea, when a student dies by suicide, the school is required to report the relevant information to the Ministry of Education in the student suicide report, which includes teachers’ observations, parental reports regarding the circumstances of death, and official education records collected by the school. Furthermore, these reports were collated as part of the national student suicide prevention policy during the abovementioned period. The evaluation items and answer format were determined through intensive discussion within the research team and feedback from teachers during the report’s development process. Additionally, specific examples of items and answers were provided in the form to simplify it and enable the teachers to understand and respond better. During the coding process, unclear answers were deciphered through discussion within the research team and confirmed by contacting the teacher directly [37, 38]. These data represent the total number of students who died by suicide in Korea during the study period. Details of the student suicide reports have been described previously [38]. The number of students who died by suicide during the study period was 654, and all cases were included in the analyses except for two students whose death dates could not be determined. Considering that Korea has compulsory education up to middle school and the dropout rate of high school in 2021 is 1.5% [39], these cases may closely represent the general characteristics of suicides among children and adolescents in Korea.

The variables used in this study were the address of the school, sex, date of death, school type, family structure, economic status, suicide method, usual concerns revealed at school, presence of a psychiatric disorder, history of suicide attempt, and history of self-injury. The teacher-rated Strengths and Difficulties Questionnaire (SDQ) [40] was used to evaluate students' emotional and behavioral status. The teacher-rated SDQ consists of Prosocial Behavior (Cronbach’s α = 0.873), Hyperactivity/Inattention (Cronbach’s α = 0.793), Peer Relationship Problems (Cronbach’s α = 0.770), Emotional Symptoms (Cronbach’s α = 0.681), Conduct Problems (Cronbach’s α = 0.638) subscales and a Total Difficulties score (Cronbach’s α = 0.837). The SDQ has been included in the database since 2018. This study was approved by the Institutional Review Board of Hallym University Sacred Heart Hospital (2021-05-015).

### Analysis

The school addresses of students who died by suicide were converted to latitude and longitude coordinates to examine the proximity of both the space and time of suicidal events, with the time of occurrence set on the day of the event. For cases with incomplete information regarding the date of death, information on the time of discovery was used. As approximately 70% of cases of adolescent suicide in Korea die by jumping from a height, the interval between the time of a suicide attempt and the time of death was expected to be short.

Clustering analysis using density-based spatial clustering of applications with noise (DBSCAN) [33, 41] was used to examine the spatiotemporal patterns of suicidal events and define the space–time clusters of suicides. Density-based clustering refers to unsupervised learning methods that identify distinctive groups or clusters in the data based on the idea that a cluster in a data space is a contiguous region of high point density, separated from other clusters by contiguous regions of low point density. The data points in the separating regions of low point density are typically considered noise/outliers [33, 41]. In particular, this method is useful when there is an outlier in the spatial information that is included in a cluster and distorted [42].

The two main conditions to be considered in DBSCAN for the derivation of clusters are the minimum number of cases to be included in the cluster and the cluster radius. In this study, the minimum number of suicide clusters was set at three. The radius of the cluster was selected by examining the change in the distance of the k-nearest neighborhood (kNN). The k value was set to three to simulate the kNN point change, which was equal to the minimum number of clustering cases. R version 4.2.2 was used for analysis and the cluster analysis was performed using the R language DBSCAN package (Hahsler et al.). The proximity among cases within the cluster is represented by the mean distance (mdis), where a lower numerical value indicates closer clustering of cases.

The final step was to compare the characteristics of clustered and non-clustered suicides. It is unreasonable to regard all the clusters derived using DBSCAN as suicide clusters. When a specific metropolitan area has a high population density, such as Seoul, suicide cases can be clustered based on regional density. Therefore, we selected a group with a high probability of suicide clusters based on a comparison of the size of the derived cluster (i.e., the number of suicide deaths) and the radius of the clusters. For group comparisons, data were examined using cross-tabulation and *t*-tests and finally included binary logistic regression analysis. In the logistic regression analysis, both the size of the region (i.e., metropolitan areas and others) and the year of suicidewere included as independent variables.

## Results

### Spatiotemporal distribution of suicide

**Fig. 1:**
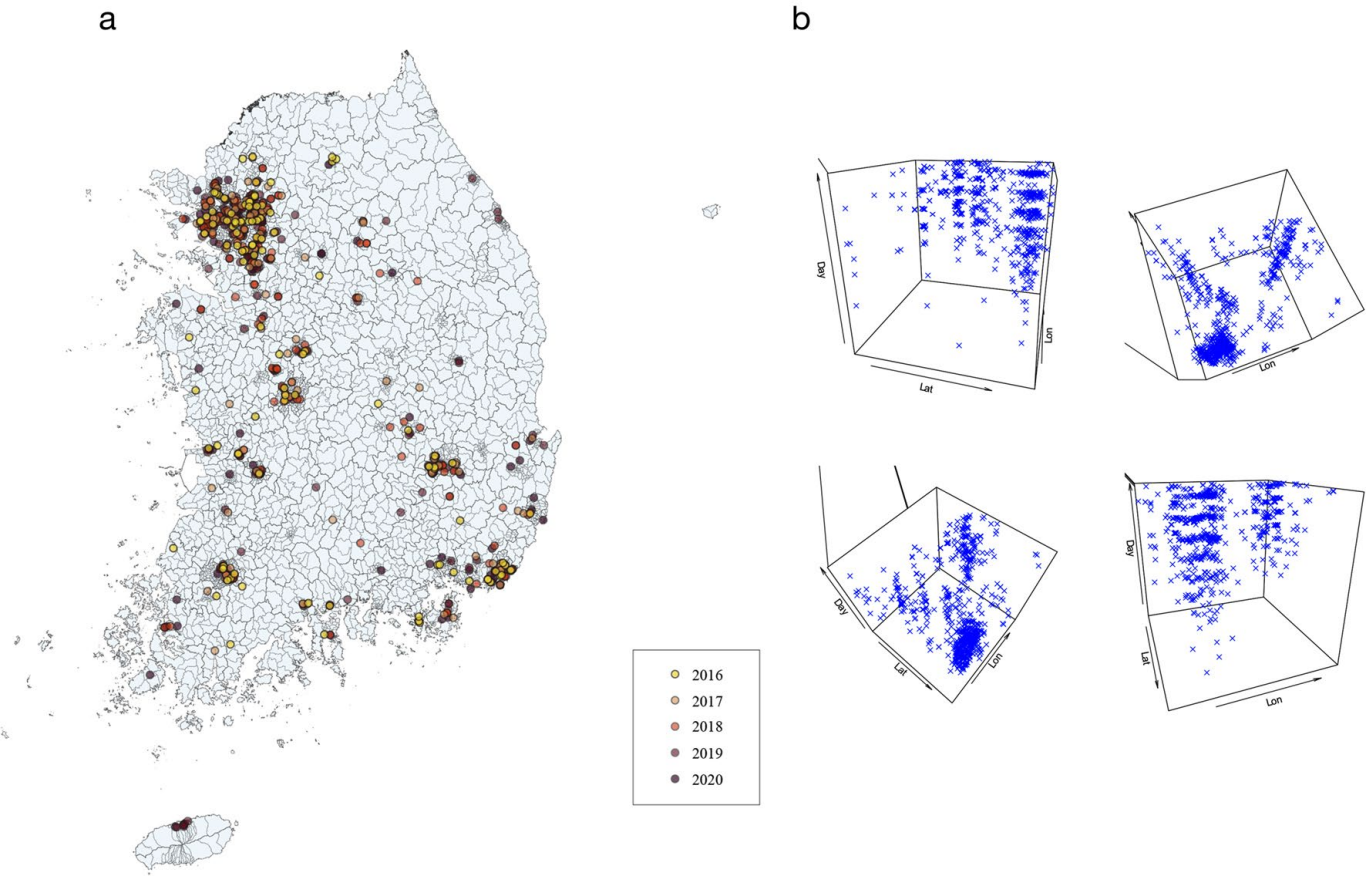
Spatiotemporal distribution of suicide cases- **a** The spatial distribution of suicide deaths marked on the map of Korea. **b** The spatiotemporal distribution of suicide cases. *Lat* latitude, *log* longitude

Figure 1 presents the spatiotemporal distribution of the suicide cases. Figure 1a shows the distribution of suicide case events on the map of South Korea, and the year of the event is also marked in a different color. Many cases were distributed around large cities with dense populations. However, even in areas with relatively sparse populations, suicide cases occur at a certain level. Figure 1b shows the results of standardizing the latitude, longitude, and time to place the case in 3-dimensional space and demonstrates that the distribution of suicide events does not occur randomly but rather clusters in a specific space–time area.

**Fig. 2:**
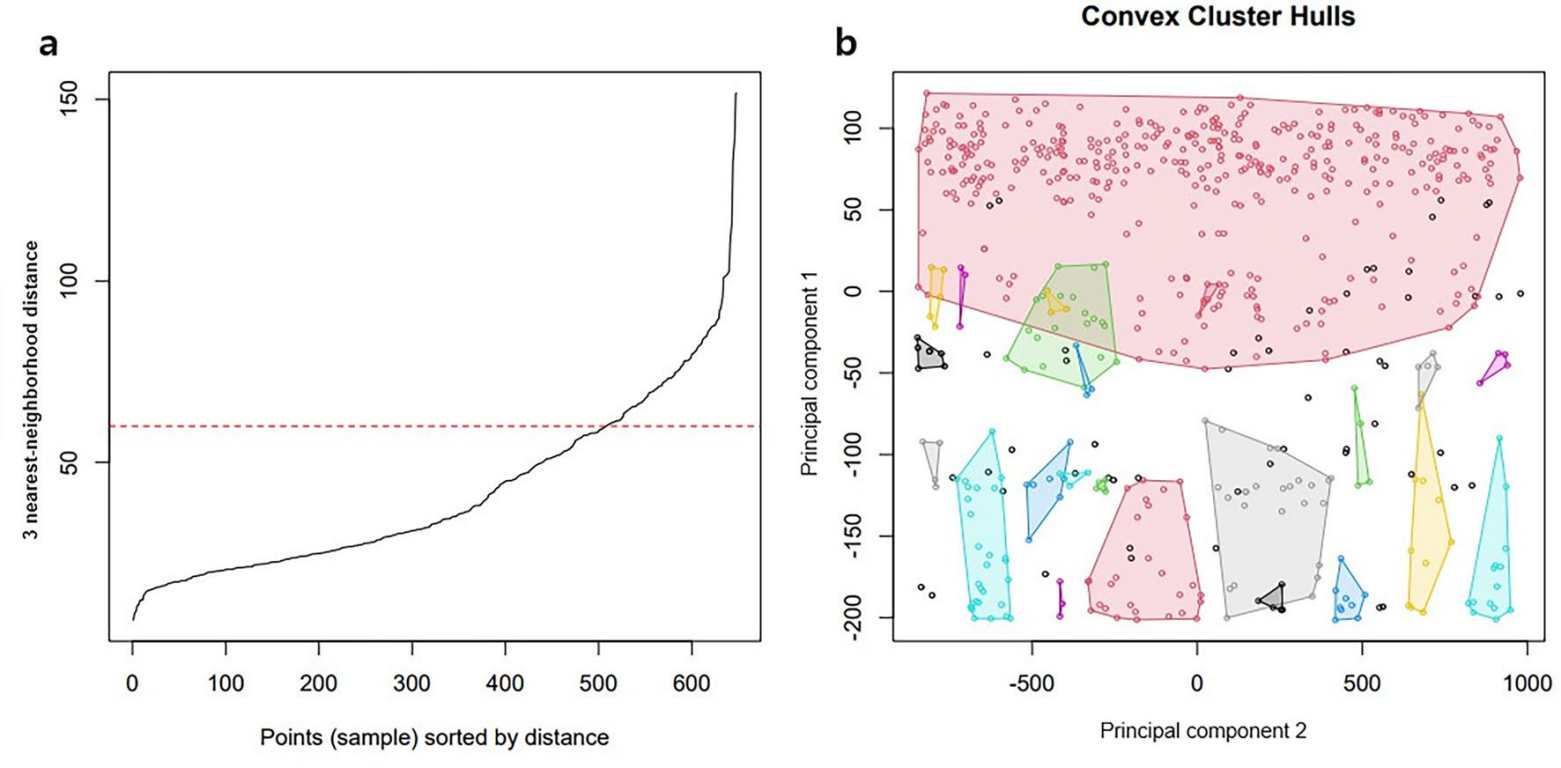
Suicide clusters converted to 2-dimensional image. *3-NN distance* 3 nearest-neighborhood distance; *PC* principal component

Figure 2a presents the results of the analyses that examined the change in the distance of the 3-nearest neighborhood to determine the criterion of the radius of the cluster prior to DBSCAN. In the figure, the knee appears around the distance of 60. Figure 2b presents the clustering results when the radius was set to 60 and the minimum number of cases belonging to a cluster was set to three. Each cluster is presented as a polygon. Outliers that did not belong to any cluster were marked as separate dots. As shown in the figure, the size of the cluster and the number of included cases varied. The largest cluster at the top of the figure reflects spatially concentrated suicide cases in densely populated areas in the Seoul metropolitan area. However, these suicides demonstrated a wide temporal distribution spanning approximately 4 years.

This led to a substantial number of cases forming the cluster (*n* = 395). Therefore, these cases cannot be regarded as meaningful spatiotemporal clusters of suicide in this study.

Table 1 presents the characteristics of the clusters derived using DBSCAN. Along with the closeness of the cluster (mdis) and number of cases in each cluster’s data distribution, the table also shows the proportion of males, high school students, middle school students, and mean age. Next, the first occurrence date, last occurrence date, longitude, and latitude of the schools attended by the students who died by suicide are presented. The latitude and longitude of the clusters were determined using the average latitude and longitude of the schools within the clusters. Significant clusters are listed in order of the smallest mdis size. Finally, they are listed based on the size of the clusters. For example, in the case of Cluster 1, which is the cluster with the most substantial spatiotemporal proximity, five cases of suicide centered on a specific area occurred within approximately 3 weeks. All patients in this cluster were high school students, and all but one were adolescent boys.

**Table 1:** Characteristics of spatiotemporal clusters detected by DBSCAN in Korea

Rank number	mdis	*n*	Proportion of males (%)	Proportion of high school students (%)	Proportion of middle school students (%)	Mean age (year)	First occurrence date of clustered suicide (year-month-day)	Last occurrence date of clustered suicide (year-month-day)	Longitude	Latitude
1	28.53698	5	80.0	100.0	0.0	18	2019-06-11	2019-07-08	128.4848	35.7728
2	29.86218	3	75.0	100.0	0.0	18	2020-08-09	2020-08-25	128.0861	37.2819
3	33.1629	3	33.3	33.3	33.3	16	2019-10-20	2019-10-27	128.9280	35.0823
3	35.83699	5	100.0	100.0	0.0	17	2020-10-12	2020-11-23	126.9308	35.9223
5	37.97212	3	33.3	100.0	0.0	19	2019-07-23	2019-09-08	129.0293	37.5960
6	40.442	5	60.0	60.0	40.0	17	2016-09-08	2016-11-05	126.8884	35.0540
7	44.4086	3	66.7	100.0	0.0	18	2018-07-02	2018-09-02	128.0431	37.2729
8	48.60928	4	25.0	75.0	25.0	18	2020-10-25	2020-12-99	128.5521	36.0997
9	48.74393	4	50.0	75.0	25.0	17	2016-02-10	2016-05-03	126.8660	35.1202
10	49.50701	4	25.0	50.0	50.0	16	2017-04-03	2017-05-18	128.4096	36.1269
11	53.87031	7	42.9	42.9	42.9	16	2020-10-10	2020-12-31	126.7332	35.0561
12	54.52508	5	60.0	80.0	20.0	17	2017-12-22	2018-03-05	129.0285	35.1898
13	55.68856	9	60.0	60.0	40.0	16	2017-04-16	2017-07-15	129.1093	35.3570
14	57.47699	3	66.7	66.7	33.3	17	2019-10-07	2019-12-05	128.0077	37.1405
15	58.3455	3	100.0	66.7	33.3	18	2019-08-03	2019-10-27	127.7446	34.9618
16	74.38226	14	42.9	92.9	7.1	18	2016-02-01	2016-06-07	128.8120	35.2468
17	74.56583	7	50.0	37.5	50.0	16	2019-09-26	2020-02-04	128.5232	35.8110
18	83.99255	10	80.0	70.0	30.0	17	2016-07-29	2016-12-05	128.6793	35.5060
19	85.55961	29	62.1	62.1	37.9	17	2020-03-25	2020-09-03	128.8434	35.4336
20	135.9051	21	38.1	71.4	19.1	18	2019-05-08	2020-04-06	126.9618	35.6613
21	140.0327	28	42.9	60.7	35.7	17	2018-08-26	2019-08-03	128.8739	35.3542
22	155.5096	25	42.3	73.1	26.9	17	2017-07-28	2018-08-13	128.6259	35.7374
23	586.5063	395	52.3	61.8	31.9	17	2016-01-03	2020-12-29	127.0052	37.2647

*mdis* mean distance

### Characteristics of defined spatiotemporal clusters for student suicide in Korea, 2016–2020

We identified 23 clusters through data analysis of 652 cases using DBSCAN. The largest cluster (class ID = 23) comprised of 395 patients. The period of the events covered approximately 5 years. As mentioned above, this cluster could result from demographic concentration, especially in the context of urban South Korea with high population density, rather than from space–time suicide clusters. Therefore, defining a significant suicide cluster that shows a remarkably high spatiotemporal adjacency.

### Comparing closeness of clusters & defining meaningful spatiotemporal clusters

**Fig. 3:**
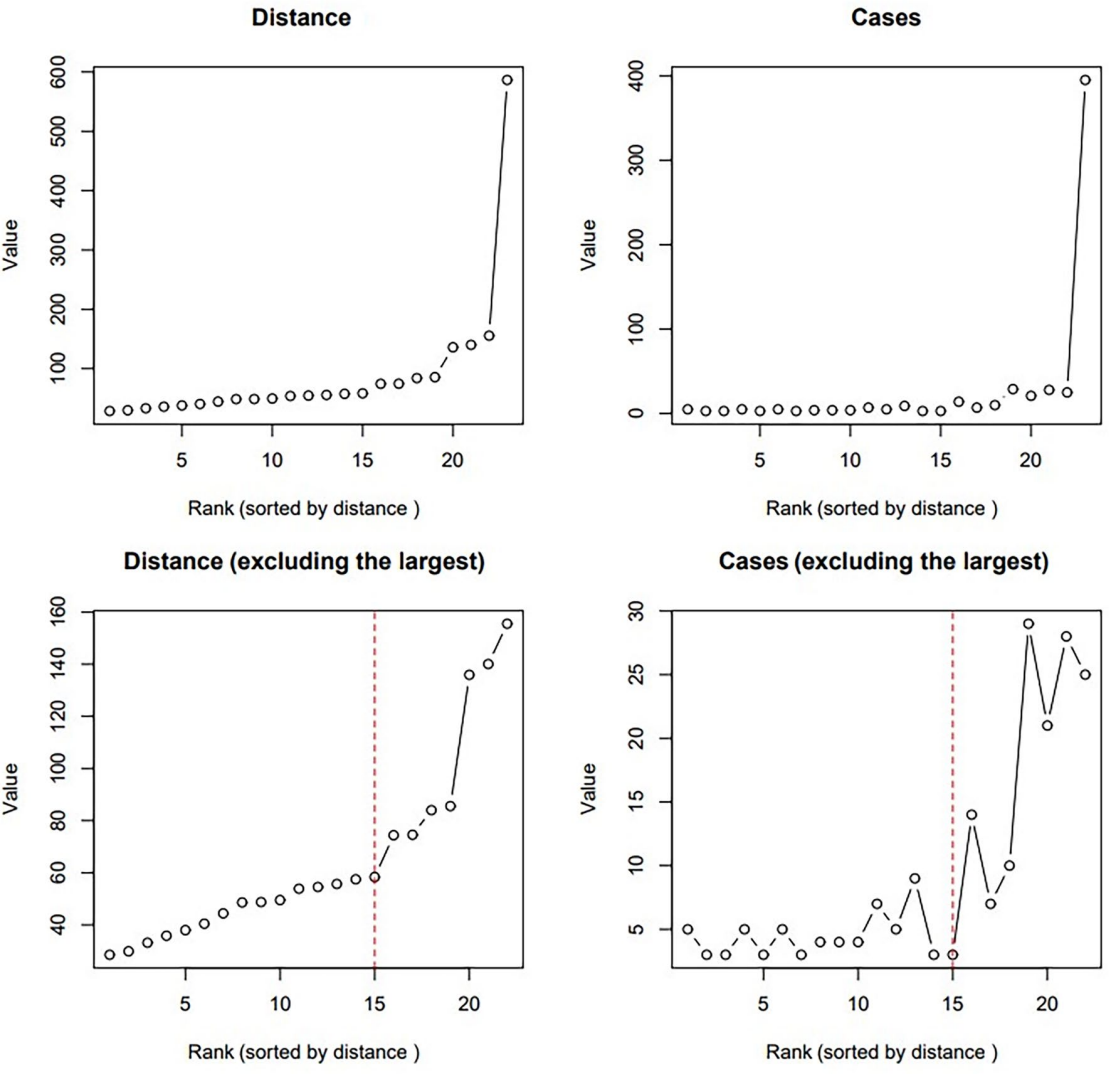
Size comparison of each cluster identified by DBCSAN-The 15th cluster is marked with a red dashed line in sequential order of distance

Figure 3 presents the results of comparing the cluster closeness (mdis) and the number of cases in the cluster data. The ranking on the horizontal axis is the result of sorting by area. The upper part of Fig. 3 presents all the clusters, and the lower part shows the figure, excluding the largest cluster. As shown in the figure, the area and number of cases rapidly increased after the 15th cluster. Based on this finding, the meaningful spatiotemporal cluster of suicide was defined as up to the 15th cluster (class ID = 12) based on the rank number. We identified 63 (9.7%) spatiotemporally clustered suicides among adolescents, with a temporal range between 7 and 59 days. In the case of spatial range, each cluster was analyzed in a polygonal form, making it difficult to precisely ascertain the average spatial area. Nonetheless, cases classified into significant clusters were predominantly within the same administrative regions. When considering the top three clusters with the highest spatiotemporal clustering (Ranks 1–3 in Table 1), the closest distance between the two suicide cases was approximately 6 km, and the greatest distance observed was approximately 32 km.

### Difference of characteristics between clustered and non-clustered suicides

Table 2 shows the comparison of the characteristics of a group that showed high spatiotemporal clustering in suicide with those of a group that did not. Chi-square analysis revealed that the characteristic that was statistically different between the two groups was economic status (χ^2^ = 9.79, df = 2, p < 0.05). The clustered suicide group was relatively low. Although no difference was observed at the stochastic significance level, participants showing clustered groupness were relatively more likely to experience peer problems. In the group without significant spatiotemporally clustered groupness, 15.1% (n = 89) reported peer problems, and in the group with clustering, 23.0% (n = 15) reported problems with peer relationships. The reported rate of psychiatric disorders was 29.7% (n = 19) in the clustering group and 40.8% (n = 231) in the other groups.

**Table 2:** Difference between clustered and non-clustered adolescent suicides: demographic and clinical characteristics

Variables			Clustering		χ^2^
			No, *n* (%)	Yes, *n* (%)	
Sex	Male		304 (51.91)	36 (57.14)	0.42
	Female		281 (48.03)	27 (42.86)	
School type	Elementary school		23 (3.93)	2 (3.17)	1.28
	Middle school		186 (31.79)	16 (25.4)	
	High school		376 (64.27)	45 (71.43)	
Family structure	Both parents		410 (70.09)	42 (66.67)	0.17
	Other^a^		175 (29.91)	21 (33.33)	
Economic state	Upper		49 (10.02)	5 (9.80)	9.79*
	Middle		333 (68.10)	25 (49.02)	
	Lower		107 (21.88)	21 (41.18)	
Suicide method	Jumping from a high place		410 (70.09)	42 (66.67)	0.39
	Hanging		146 (24.96)	18 (28.57)	
	Other		29 (4.96)	3 (4.76)	
Usual concerns^b^	Family problems	No	368 (62.69)	35 (54.69)	1.25
		Yes	219 (37.31)	29 (45.31)	
	Academic problems	No	375 (63.56)	42 (64.62)	0.00
		Yes	215 (36.44)	23 (35.38)	
	Peer problems	No	499 (84.86)	50 (76.92)	2.20
		Yes	89 (15.14)	15 (23.08)	
History of psychiatric disorder	No		355 (59.19)	45 (70.31)	1.92
	Yes		231 (40.81)	19 (29.69)	
History of suicide attempt	No		542 (92.49)	59 (93.65)	0.00
	Yes		44 (7.51)	4 (6.35)	
History of self-harm	No		516 (88.21)	56 (88.89)	0.00
	Yes		69 (11.79)	7 (11.11)	

*p < 0.05

^a^Single parent, family of grandparents, orphanage, etc.

^b^Usual concerns revealed at school within 1 year prior to the death of a student who died by suicide

Table 3 presents the results of the group comparisons using the SDQ. The results of the *t-*tests indicated that there were no statistically significant differences between the two groups for SDQ total and subscale scores.

**Table 3:** Difference between clustered and non-clustered adolescent suicides: SDQ Total and subscale scores

SDQ		Clustering		*t*
		No, Mean (SD)	Yes, Mean (SD)	
Strengths	Prosocial behaviors	5.95 (2.57)	6.40 (2.84)	− 0.90
Difficulties	Hyperactivity	2.61 (2.21)	2.35 (2.49)	0.61
	Emotional problems	2.29 (2.29)	2.34 (2.34)	− 0.01
	Conduct	1.23 (1.33)	1.81 (2.30)	− 1.52
	Peer problems	2.03 (2.03)	1.97 (2.05)	0.19
Total difficulties		8.06 (5.32)	8.43 (6.67)	− 0.33

In Table 4, the binary logistic regression analysis results are presented, with the highly clustered group being the outcome variable and the non-clustered cases being the reference group. The demographic and clinical characteristics that were found to significantly differ based on group included economic status (e.g., poverty) and the presence of a psychiatric disorder (*p* < 0.05). As the economic level decreases (indicative of poverty), there is an increased tendency for spatiotemporal clustering. However, the less likely the cases included reported psychiatric disorders, the more likely they were to be in a highly clustered group. Groups that reported peer problems had a higher likelihood of being highly clustered, even though the statistical significance of this result was low (*p* < 0.10).

**Table 4:** Result of multivariate logistic regression analysis

Variables		*b*	*SE*	*z*	*p*-Value
(Intercept)		− 2.57	0.93	− 2.77	0.000
Sex	(Ref. = male)				
	Female	− 0.05	0.29	− 0.18	0.802
School type	(Ref. = Elementary School)				
	Middle school	− 0.10	0.81	− 0.13	0.184
	High school	0.30	0.78	0.39	0.610
Family structure	(Ref. = both parents)				
	Other	0.17	0.30	0.58	0.700
Economic status	(Ref. = non-poverty)				
	Lower	0.70	0.33	2.14	0.040*
Usual concerns: family problems	(Ref. = No)				
	Yes	0.24	0.32	0.77	0.434
Usual concerns: peer problems	(Ref. = No)				
	Yes	0.63	0.38	1.67	0.067°
Usual concerns: Academic problems	(Ref. = No)				
	Yes	− 0.08	0.30	− 0.26	0.871
History of self-harm	(Ref. = No)				
	Yes	− 0.18	0.47	− 0.39	0.575
History of psychiatric disorder	(Ref. = No)				
	Yes	− 0.72	0.34	− 2.13	0.042*
Year of suicide	(Ref. = 2016)				
	Year_2017	0.66	0.45	1.46	0.121
	Year_2018	− 0.81	0.58	− 1.39	0.153
	Year_2019	0.37	0.46	0.79	0.466
	Year_2020	0.67	0.44	1.52	0.132

**p < 0.01, *p < 0.05, °*p* < 0.10

## Discussion

This study identified space–time clusters of cases of adolescent suicide using DBSCAN based on Korean student suicide data from 2016 to 2020. As a result, 9.7% (*n* = 63) corresponded to the space–time suicide cluster, and each cluster consisted of 3–9 suicide events and suicides temporally occurring between 7 and 59 days and corresponded to the distances between suicide cases within the top three most concentrated clusters, ranging from 6 to 32 km spatially. The suicide cluster group had low economic status and fewer psychiatric disorders compared to the non-clustered group. To the best of our knowledge, this is the first study to use latitude and longitude for spatial analysis and exact suicide dates for temporal analysis in the clustering of adolescent suicides, and it uses narrower spatiotemporal units of analysis than previous studies using DBSCAN without pre-setting spatiotemporal parameters.

### Clustered suicides of adolescents in Korea during 2016–2020

In this study, 9.7% of adolescent suicides were classified into spatiotemporal suicide clusters, which was a higher percentage than previously reported. This increase could be attributed to differences in the analytic methods. The current findings suggest that interrelated suicides may be more frequent than expected in adolescents [5]. However, this study statistically identified spatiotemporal suicide clusters but did not confirm that suicides within clusters were actually related to suicides. Suicides that occurred within similar time periods in similar locations could have been classified into this space–time suicide cluster, even if there was no real connection. Future research should include a detailed case study of the suicide cases in these clusters.

The mechanisms leading to suicide clusters include social transmission, particularly person-to-person transmission and the media [5, 6, 18]. In addition, clustered suicide occurs through perceptions that suicidal behavior is widespread and assortative, leading to susceptible young people being likely to socialize with at-risk peers, and the social cohesion of the peer group contributes to the spread of ideas and attitudes [5, 6]. The effect of suicide clusters on schools is usually profound, and the early identification of suicide clusters and initiation of appropriate interventions is critical for preventing subsequent suicides. This study suggests that once an adolescent died by suicide, close monitoring and intervention may be needed to prevent subsequent suicides for about 2 months.

### Characteristics of clustered adolescent suicides in Korea: Comparison with previous studies

Several features of the clustered adolescent suicides in this study were similar to the socioeconomic characteristics of previously identified clustered suicides. Previous studies have identified deprivation [31, 43], poverty [22, 23, 43–45], and geological isolation [25, 30, 46] as significant risk factors for clustered suicide. In this study, economic status was lower among the clustered suicides than the non-clustered suicides in both the chi-square test and logistic regression analysis, which mirrors the results of previous studies.

In previous studies, young men were more frequently included in the clustered suicide groups than were young women [24, 47, 48]. However, this finding has not been replicated in other population-based studies targeting young adults and adolescents [5, 11, 25]. Similarly, there was no difference in the gender ratio between clustered and non-clustered suicides in our study, the first to report the gender characteristics of clustered suicides in Korean adolescents. This could be due to differences in the analytical method (DBSCAN) used to identify suicide clusters between this study and previous studies. Furthermore, 654 suicides were included in the analysis, which is fewer than in previous studies; this could have potentially influenced the results. Hence, future studies that target a larger number of suicides over an extended period are needed.

Regression analysis revealed that the clustered suicide groups had fewer psychiatric disorders than the non-clustered suicide group. This differs from previous findings and suggests that psychiatric history is a risk factor for clustered suicides [6]. However, it should be noted that the assessment of psychiatric disorders among students who died by suicide was based on parental reports after suicide rather than the direct application of standardized diagnostic tools, thus potentially failing to adequately capture the frequency of psychiatric disorders. Even if the students had clinically diagnosed psychiatric disorders, they may not have visited hospitals because of negative perceptions associated with mental health or that parents did not accurately report due to concerns about potential disadvantages the students might face at school. Additionally, no statistically significant differences were observed regarding the presence of psychiatric disorders between the two groups in the chi-square test. Given the limited number of participants, further research is necessary to address these findings.

Another distinctive characteristic of clustered group was their low economic status, which is consistent with previous studies [18, 22, 23, 31, 43–45]. However, earlier studies have not clarified the relationship between socioeconomic status and suicide clustering. In some studies [22], low economic status has been suggested as a proxy for factors associated with the clustering of suicides, such as limited access to mental health treatment. Since limited information was collected from each participant, our study could not clearly explain the underlying mechanism. Considering the multidimensional risk factors of adolescent suicide [49], and the general social stigma against psychiatric disorders in South Korea [50], having a low economic status might also decrease help-seeking behavior for the early detection of mental health problems of clustered suicide adolescents in Korea.

Although differences in peer problems were a non-significant trend (*p* < 0.10) between the groups, the clustered suicide group reported more peer problems than the non-clustered group. When examining each case of clustered suicide, it is apparent that the students included in the clustered suicide did not exhibit considerable vulnerability to suicide on a personal level. Considering the other characteristics mentioned above, this finding may be because they grew up in economically disadvantaged households with vulnerable support systems, delayed their development of introspection and help-seeking behaviors, and lacked resilience, leading to their immersion in peer relationships.

In summary, by using DBSCAN to analyze clustered adolescent suicides in Korea, we found a higher rate (9.7%) than that reported in previous studies. Moreover, the temporal range for the clustered suicides identified was within 2 months. These suicides were characterized by lower economic status, which is consistent with previous studies [22, 23, 43–45]. Our study differs from previous studies in that we used a methodology that did not use a specific window, providing a basis for identifying the critical time and regions for subsequent adolescent suicide prevention.

### Limitations

This study has several limitations. First, suicide cases among adolescents used in our study only included those reported by schools; thus, out-of-school adolescents were excluded. Second, our study exclusively focused on Korean students who died by suicide over 5 years, resulting in a limited sample size. This is because our study was a secondary analysis of data collected during a limited period, 2016–2020, as part of a suicide prevention policy in Korea. Correspondingly, given the exclusive focus on Korean adolescents, the distinct attributes of suicide may be influenced by national and cultural contexts, impeding the generalization of this study’s outcomes to diverse international settings. Third, we defined clustering as involving a minimum of three suicides; thus, cases in which two consecutive suicides occurred in a spatiotemporal context similar to clustered suicides were not included. Fourth, we did not account for factors that could link adolescents who died by suicide, even when not in geographically similar spaces, such as the Internet or social network services. Therefore, clustered suicides among adolescents might not have been adequately identified. Finally, the geographic data employed in this study were derived from school addresses rather than the residential addresses of adolescents who died by suicide, consequently failing to accurately reflect the specific locations of suicide incidents. However, Korean students are assigned to schools through a system known as the school district [51], wherein the proximity of a student’s residence serves as the paramount criterion for school assignment. Therefore, the addresses of the schools utilized in our research can indirectly represent the actual places of residence, and this window is much narrower than the previous studies that used county-level data. Additionally, in the Korean context, the living environment and peer groups of adolescents are often organized on a school-based scale, thereby highlighting the significance of the findings of this study.

## Conclusion

In this study, the clustering of suicides was analyzed using a novel analytical method (DBSCAN) that differs from previous studies. As a result, a higher prevalence of clustered suicides (9.3%) among the total population of adolescent suicides was observed compared to previous research. Also, this study suggests that once an adolescent suicide occurs, close monitoring and intervention is needed for approximately 2 months to prevent subsequent suicides. Notably, this clustering was pronounced among those with low social-economic status. Future research using DBSCAN needs to involve a larger sample of adolescents from various countries. Clarifying the underlying mechanisms behind clustered suicides among adolescents could help enhance efforts to prevent adolescent suicide.

## Data Availability

The data from the Korean student suicide reports used in our study will not be publicly available. Interested parties can obtain the data by contacting the corresponding author (HJH) through a reasonable request.

## Abbreviations

SDQ: Strengths and difficulties questionnaire
DBSCAN: Clustering analysis using density-based spatial clustering of applications with noise
kNN: K-Nearest neighborhood
mdis: Mean distance
